# Investigating insecticide resistance and knock-down resistance (*kdr*) mutation in Dielmo, Senegal, an area under long lasting insecticidal-treated nets universal coverage for 10 years

**DOI:** 10.1186/s12936-018-2276-7

**Published:** 2018-03-22

**Authors:** Omar Thiaw, Souleymane Doucouré, Seynabou Sougoufara, Charles Bouganali, Lassana Konaté, Nafissatou Diagne, Ousmane Faye, Cheikh Sokhna

**Affiliations:** 1 UMR Vecteurs-Infections Tropicales et Méditerranéennes (VITROME), Campus International UCAD-IRD Hann Maristes, Dakar, Senegal; 2 Laboratoire d’Ecologie Vectorielle et Parasitaire (LEVP), Faculté des Sciences et Techniques (FST), Université Cheikh Anta Diop (UCAD), Dakar, Senegal

**Keywords:** LLINs, Insecticide resistance, *kdr*, Metabolic resistance, *Anopheles arabiensis*, *Anopheles coluzzii*, *Anopheles gambiae* s.s., Vector control, Dielmo, Senegal

## Abstract

**Background:**

The use of insecticides, through indoor residual spraying and long-lasting insecticide-treated nets (LLINs), is essential to control malaria vectors. However, the sustainability of these tools is challenged by the spread of insecticide resistance in *Anopheles* mosquitoes. This study was conducted to assess the susceptibility to insecticides and to determine the resistance mechanisms in malaria vectors in Dielmo, a rural area of western Senegal where LLINs were introduced a decade ago.

**Methods:**

CDC bottle bioassays were used to determine the susceptibility of 2–5 day-old unfed *Anopheles gambiae* s.l. females to alphacypermethrin (12.5 µg/bottle), deltamethrin (12.5 µg/bottle), etofenprox (12.5 µg/bottle), lambdacyhalothrin (12.5 µg/bottle), permethrin (21.5 µg/bottle), DDT (100 µg/bottle), bendiocarb (12.5 µg/bottle), pirimiphos-methyl (20 µg/bottle) and fenitrothion (50 µg/bottle). The involvement of glutathione-*S*-transferases (GSTs) in insecticide resistance was assessed using a synergist, etacrynic acid (EA, 80 µg/bottle). Polymerase chain reaction (PCR) was used to investigate the presence of ‘knock-down resistance (*kdr*)’ mutation and to identify sibling species within the *An. gambiae* complex.

**Results:**

CDC bottle bioassays showed that mosquitoes were fully susceptible to lambdacyhalothrin, bendiocarb and fenitrothion. Overall, mortality rates of 97, 94.6, 93.5, 92.1, and 90.1% were, respectively, observed for permethrin, deltamethrin, pirimiphos-methyl, etofenprox and alphacypermethrin. Resistance to DDT was observed, with a mortality rate of 62%. The use of EA significantly improved the susceptibility of *An. gambiae* s.l. to DDT by inhibiting GSTs (*p *= 0.03). PCR revealed that *Anopheles arabiensis* was the predominant species (91.3%; IC 95 86.6–94%) within *An. gambiae* complex from Dielmo, followed by *Anopheles coluzzii* (5.4%; IC 95 2.7–8.1%) and *Anopheles gambiae* s.s. (3.3%; IC 95 0.6–5.9%). Both 1014F and 1014S alleles were found in *An. arabiensis* population with frequencies of 0.08 and 0.361, respectively, and 0.233 and 0.133, respectively in *An. coluzzii*. In *An. gambiae* s.s. population, only *kdr* L1014F mutation was detected, with a frequency of 0.167. It was observed that some individual mosquitoes carried both alleles, with 19 specimens recorded for *An. arabiensis* and 2 for *An. coluzzii*. The presence of L1014F and L1014S alleles were not associated with resistance to pyrethroids and DDT in *An. arabiensis*.

**Conclusions:**

The co-occurrence of 1014F and 1014S alleles and the probable involvement of GSTs enzymes in insecticide resistance in *An. gambiae* s.l. should prompt the local vector programme to implement non-pyrethroid/DDT insecticides alternatives.

## Background

The constant and significant decrease of malaria incidence reported during recent years has instilled a hope that malaria elimination is a feasible objective in several endemic areas. The current situation is driven by the combination of different malaria control strategies delivered through National Malaria Control Programmes (NMCPs) supported by financial partners [1]. The main tools used are artemisinin-based combination therapy (ACT) to treat *Plasmodium* infection, indoor residual spraying (IRS) and long-lasting insecticide-treated nets (LLINs) to control malaria vectors. Of these, the use of LLINs remains the most effective strategy to control malaria in endemic areas [2, 3]. During the period 2001–2015, malaria control tools have helped to prevent 633 million cases, and LLINs have been allocated a 69% share of that estimation, in contrast to 21% for ACT and 10% for IRS. In sub-Saharan Africa, 53% of the population at risk slept under LLINs [1]. However, the development of insecticide resistance in *Anopheles* populations is threatening the effectiveness of LLINs. Some studies have shown that resistant mosquitoes can blood-feed effectively despite the use of LLINs, even when net integrity is not compromised [4–6].

Insecticide resistance has been associated with the use of pesticides for agricultural purposes and for malaria vector control strategies, through IRS and LLINs [7, 8]. Pyrethroid resistance is commonly reported; in 2014, it was recorded in three-quarters of malaria-endemic countries [1]. This situation is alarming as pyrethroids are the only insecticides approved by the World Health Organization (WHO) for LLIN impregnation. Global efforts deployed to control malaria vectors through LLINs could be jeopardized by pyrethroid resistance. Target site resistance and metabolic resistance are the two major mechanisms typically assumed to be responsible for mosquito resistance to insecticides [9]. The voltage-gated sodium channel (VGSC), located in the insect’s nervous system, is the target site for pyrethroids and DDT. Mosquitoes resistant to these insecticides exhibit some modification of their VGSC, due to a mutation of the gene encoding this protein [10]. This target site resistance, better known as ‘knock-down resistance*’* (*kdr*), results from a substitution of a leucine amino acid at codon 1014 by a phenylalanine (L1014F or *kdr*-*west*) or by a serine (L1014S or *kdr*-*east*). *Kdr*-*west* and *kdr*-*east* mutations were described for the first time in West [11] and East [12] Africa, respectively. However, recent findings suggest that the circulation of L1014F and L1014S alleles is not geographically limited and both mutations are today found in West and East African countries [13, 14].

Metabolic resistance is due to changes in the mosquito’s enzyme systems which results in rapid detoxification of the insecticide preventing it from reaching the site of action within the mosquito. Several families of enzymes, including cytochrome P450 monooxygenases (P450s), glutathione-*S*-transferases (GSTs) and carboxylesterases, are involved in mosquito metabolic resistance to insecticides [9, 15–27]. Some chemical compounds, such as piperonyl butoxide (PBO), *S*-*S*-*S*-tributylphosphorotrithioate (DEF) and etacrynic acid (EA), can be used as synergists to inhibit P450s, carboxylesterases and GSTs, respectively [28]. To deal with insecticide resistance, particularly in areas where insecticide-based tools have been implemented for several years, it is relevant to monitor the dynamics and the level of *Anopheles* susceptibility to insecticide while exploring insecticide resistance mechanisms. Dielmo village, in rural Senegal, has been under LLIN universal coverage since 2008. The use of LLINs in this setting has enabled the control of *Anopheles gambiae* sensu lato (s.l.) and *Anopheles funestus*, the main malaria vectors, and to reduce drastically malaria incidence; occasional malaria cases observed were attributed to a lack of LLIN use [29].

Notwithstanding the collapse of malaria incidence in Dielmo through the long-term use of LLINs, there is little information on insecticide resistance in malaria vectors. The main purpose of this work was to investigate insecticide resistance in *An. gambiae* s.l. to insecticides, a decade since the introduction of LLINs in Dielmo.

## Methods

### Study area

The study was conducted in Dielmo, which is located in the Fatick region, 280 km from Dakar, in a Soudan-type climate area. Since 1990, a regular epidemiological survey has been conducted in this setting to understand the epidemiology of malaria [30]. In Dielmo, malaria vector control has always been based on LLIN universal coverage. This strategy was implemented in July 2008. Since then, it has been maintained through three LLIN general renewals, in 2011, 2014 and 2016. In Dielmo, IRS has never been introduced. In 2015, there were approximately 481 inhabitants distributed in 42 concessions. The rainy season usually lasts from July to October. In 2015, the first rains were recorded in July. August was the wettest month, followed by September, with respective monthly rainfalls of 262 and 225 mm. In October, rainfall of only 94 mm was recorded. Overall, in 2015, annual rainfall was estimated at 784 mm and the mean temperature ranged from 22 to 35 °C. The villagers are millet and groundnut farmers, but during the dry season, market gardening is practised along the swampy bank of a small permanent river, the Nema, around which market-garden wells or *ceanes* are dug for watering. In Dielmo, commercial pesticides including organophosphates (Dimethoate^®^, Pyrical 480EC^®^), organochlorine and pyrethroids, most often in combination (Callifan Super 40EC^®^), are used for agricultural purposes.

### *Anopheles* immature stages collection and mosquito rearing

**Fig. 1:**
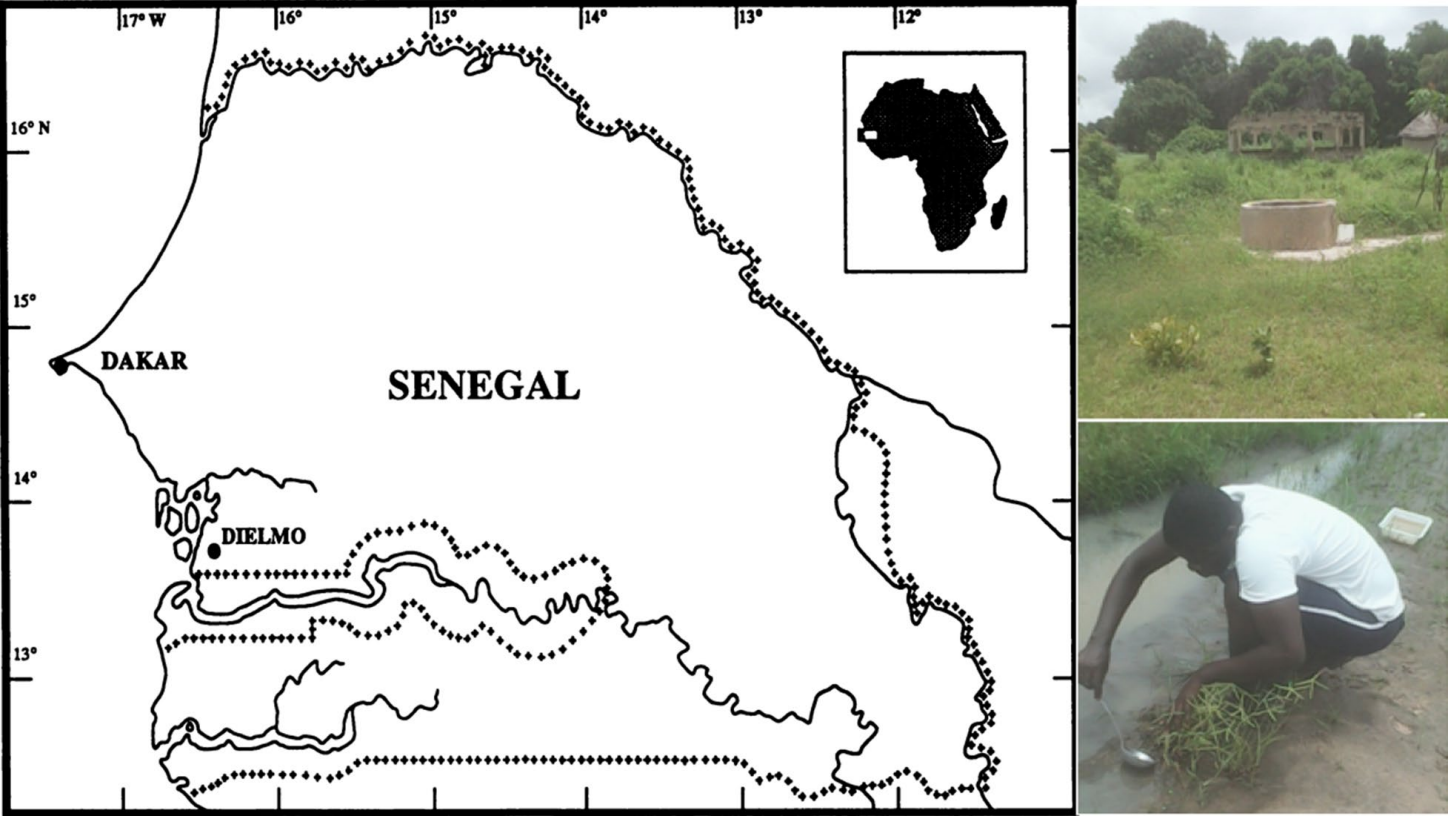
Map showing the location of Dielmo (study area), its aspect in the rainy season and one anopheline breeding site

Surveys were carried out in September 2015 in Dielmo. During the study period, five breeding sites were identified in and around the village and were all used to collect *Anopheles* larvae and pupae. These breeding sites consisted of fresh water with vegetation, especially from the *Gramineae* family (Fig. 1). Larval collections were pooled in sampling containers and transferred to a local insectary for rearing. Larvae were fed with fishmeal (Tetramin Baby^*®*^). Pupae were daily collected and introduced into rearing cages using small plastic cups. At emergence, mosquito adults were fed using absorbent cotton soaked with 10% sucrose solution.

### *Anopheles* susceptibility to insecticides

The Centers for Disease Control and Prevention (CDC) bottle bioassays was used to assess *Anopheles* susceptibility to insecticides. Insecticides were provided by CDC in lyophilized forms or in hyper-concentrated formulations. In the laboratory, reconstitution or dilution was performed using acetone to obtain stock solutions of which 1 mL contained the diagnostic dose of the insecticide (expressed in µg/bottle). Stock solutions were then stored at 4 °C in foil-wrapped Falcon tubes before being transported into the field using an electric icebox. Wheaton glass (250 mL) bottles were coated using 1 ml of insecticide solution as described in the CDC protocol [28]. Five pyrethroid insecticides (Alphacypermethrin 12.5 µg/bottle, Deltamethrin 12.5 µg/bottle, Etofenprox 12.5 µg/bottle, Lambdacyhalothrin 12.5 µg/bottle and Permethrin 21.5 µg/bottle), one organochlorine (DDT 100 µg/bottle), one carbamate (Bendiocarb 12.5 µg/bottle) and two organophosphates (Pirimiphos-methyl 20 µg/bottle and Fenitrothion 50 µg/bottle) were tested. Bioassays were carried out on 2–5 day-old unfed females using the CDC bottle protocol [28, 31, 32]. Each insecticide molecule was tested in four replicates using 16–25 female mosquitoes for each replicate; a control bottle with 20–25 female mosquitoes was used in parallel. The temperature in the field testing room was 25 ± 3 °C and the relative humidity was 75 ± 10%. Mortality was recorded at 15-min intervals until insecticide-diagnostic time (DT) was reached. Mortality at DT represents the most critical value in CDC bottle bioassays and thus, in this study any 24-h holding period for mortality recording was not taken into account. The DT was 30 min for all insecticides except DDT which had a DT of 45 min [28]. A mosquito was considered dead if it could not stand or fly in coordinated way, especially when the bottle was gently rotated while making the count. When the DT of the insecticide being tested elapsed, mosquitoes were transferred to holding cartons and survivors were collected using an aspirator and then killed with chloroform. The number of dead mosquitoes for all replicates of each test was used to determine percentage mortality. Finally, both surviving and dead mosquitoes were individually stored in numbered Eppendorf tubes containing silica gel. A random sample of dead mosquitoes and survivors were used for species identification and the detection of *kdr* gene.

### Detection of detoxification enzymes using synergist

In case mosquitoes displayed mortality rate below resistance threshold after exposure to DDT, complementary bioassays with synergist EA (80 µg/bottle) were performed according to CDC protocol [28], using another mosquito sample from the population previously tested. A batch of 100–125 unfed females was exposed to one bottle coated with EA (EA-exposed mosquitoes). Another batch with the same number of mosquitoes was used as a control and was exposed to one acetone-coated bottle (non-EA-exposed mosquitoes). After 1 h of exposure, mosquitoes from each batch were separately released into rearing cages. Then, EA-exposed and non-EA-exposed mosquitoes were in parallel exposed to DDT (100 µg/bottle) according to standard CDC bottle bioassay as above. Data comparisons were made between mortality rates from EA-exposed and non-EA-exposed mosquitoes.

### Species identification and detection of *kdr*-*w* and *kdr*-*e* mutations

CTAB Method was used for extracting genomic deoxyribonucleic acid (DNA) from entire mosquitoes. The one-step polymerase chain reaction (PCR) technique using intentional mismatch primers (IMPs) described by Wilkins et al. [33] was used to identify the sibling species of the *An. gambiae* complex. L1014F (*kdr*-*w*) and L1014S (*kdr*-*e*) mutations were investigated using also IMP PCR primers [34]. A volume of 1–2 µL of DNA template was added to 23–25 µL PCR Master Mix containing *Taq* DNA Polymerase, Microbial DNA-free water, 5X Green GoTaq Buffer, 2.0–2.5 mM dNTP, 25 mM MgCl_2_ and 2.5–25 pmol/µL of specific primers. PCR amplification conditions in a Bio-Rad^®^ Thermocycler were as follows: 95 °C for 5 min for 1 cycle, followed by [95 °C for 30 s, 58 °C (or 57/59 °C for *kdr*-*e*/*kdr*-*w*) for 30 s and 72 °C for 30 s] for 30 cycles (or 35 cycles for *kdr*-*e* and *kdr*-*w*), followed by 1 cycle of 72 °C for 5 min. PCR amplicons were migrated on 2% agarose gel mixed with fluorescent Gel Red DNA stain (BIOTIUM^®^). Specific target bands were visualized under UV light using a Bio-Rad^®^Gel Doc XR connected to computer running software (Quantity One, 4.6.6 Basic Version).

### Data interpretation and statistical analysis

Percentage mortality was interpreted according to WHO criteria [35] to determine susceptibility status. Pearson’s Chi squared test was used to compare mortality rates. Allelic association studies were conducted using Fisher exact test and odds ratio test. A p value less than 5% was considered as significant. All statistical analyses and graphs were made using R software version 3.4.1 [36].

## Results

### Susceptibility to insecticides

**Fig. 2:**
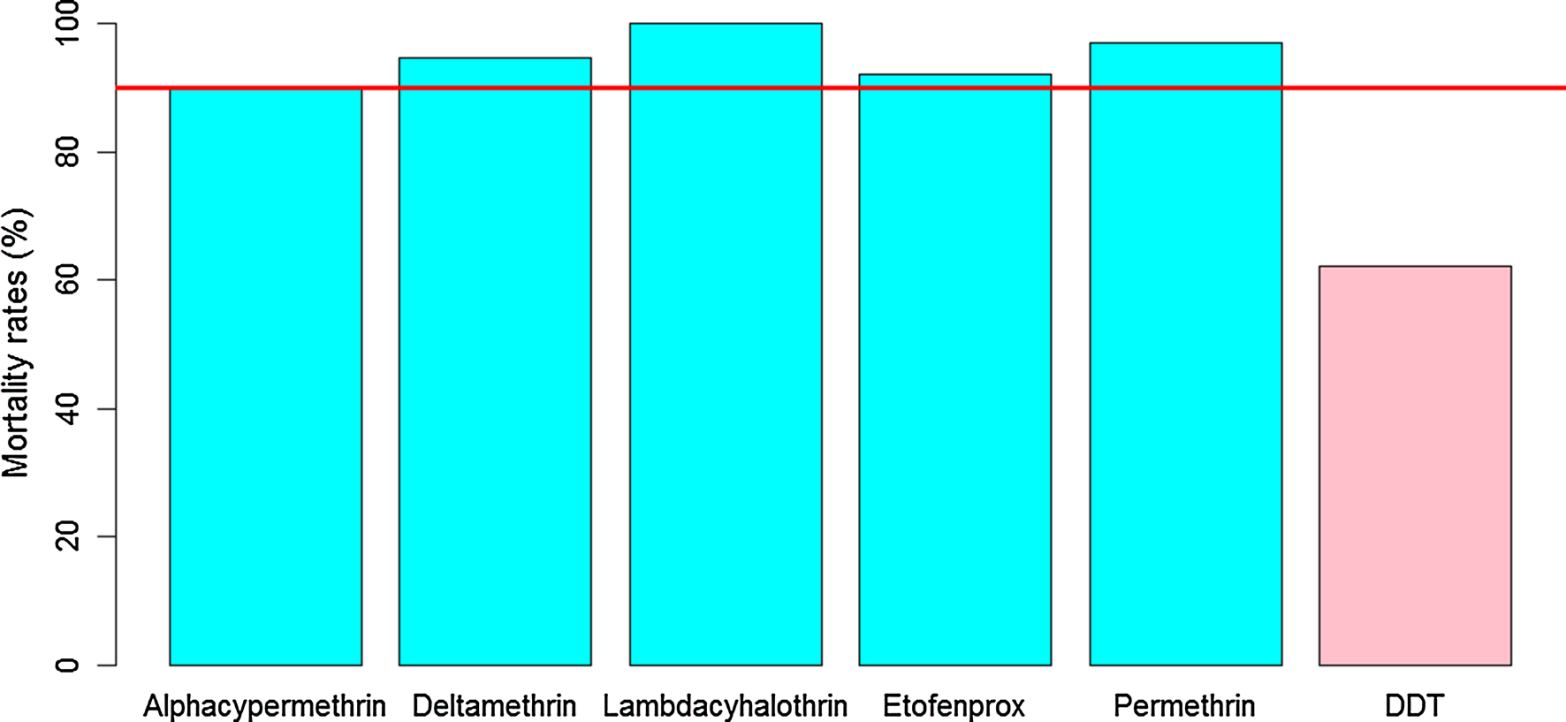
Mortality rates of *Anopheles gambiae* s.l. from Dielmo, after exposure to pyrethroids (alphacypermethrin 12.5 µg/bottle, deltamethrin 12.5 µg/bottle, etofenprox 12.5 µg/bottle, lambdacyhalothrin 12.5 µg/bottle, permethrin 21.5 µg/bottle) and DDT 100 µg/bottle (organochlorine). Percentage mortality was plotted after exposure at DT of each insecticide. The horizontal line indicates a 90% resistance threshold according to WHO criteria. Pyrethroids are represented by blue bars and organochlorine by pink bars

**Fig. 3:**
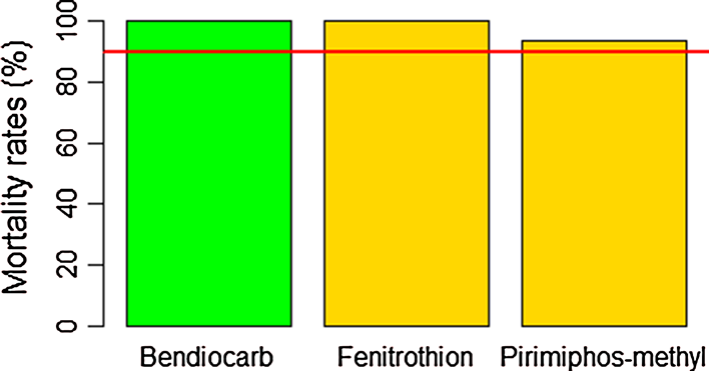
Mortality rates of *Anopheles gambiae* s.l. from Dielmo, after exposure to one carbamate (bendiocarb 12 µg/bottle) and organophosphorous insecticides (pirimiphos-methyl 20 µg/bottle and fenitrothion 50 µg/bottle). Percentage mortality was plotted after exposure at DT of each insecticide. The horizontal line indicates a 90% resistance threshold according to WHO criteria. Carbamate is represented by green bar and organophosphates by gold bars

A total of 834 *An. gambiae* s.l. female mosquitoes were tested with 9 insecticides. In the group of pyrethroids (Fig. 2), full mortality (100%) was only observed with lambdacyhalothrin (n = 91), after 30 min of exposure. Resistance was suspected with permethrin, deltamethrin, etofenprox, and alphacypermethrin, with mortality rates of 97% (n = 67), 94.6% (n = 110), 92.1% (n = 101), and 92.1% (n = 101), respectively, after 30 min of exposure (Fig. 2). *Anopheles gambiae* s.l. was resistant to DDT, with a mortality rate of 62% (n = 108), after 45 min of exposure (Fig. 2). *Anopheles gambiae* s.l. showed full mortality with fenitrothion (n = 74) and a probable resistance to pirimiphos-methyl with a mortality rate of 93.5% (n = 92) after 30 min. Full susceptibility (n = 100) was observed with bendiocarb (carbamate), after 30 min of exposure (Fig. 3). Globally, 205 control mosquitoes were used during the bioassays and no mortality was observed in control bottles.

### Detection of detoxification enzymes with synergist

**Fig. 4:**
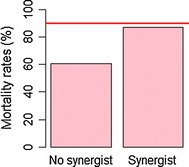
Effect of etacrynic acid (EA 80 µg/bottle) on DDT-resistant *Anopheles gambiae* s.l. from Dielmo. Mosquitoes from two separate batches were exposed to one acetone-coated bottle (no synergist) and to another EA-coated one (synergist) for 1 h, before being both tested with DDT-treated bottles for 45 min (DDT DT). Percentage mortality was plotted after exposure at DT of each insecticide. The horizontal line indicates a 90% resistance threshold according to WHO criteria

In the study, only EA (80 µg/bottle) which inhibits GSTs, was used as synergist for DDT-resistant mosquitoes. The prior exposure of mosquitoes to EA before testing with DDT increased significantly (χ^2^ = 4.6, df = 1, *p *= 0.03) the mortality rate (87%, n = 113) compared to the control mosquitoes exposed to only DDT (60.97%, n = 97). However, the mortality rate after pre-exposure to EA was still below the resistance threshold (Fig. 4).

### Species identification and detection of *kdr*-*w* and *kdr*-*e* mutations

A total of 280 *An. gambiae* s.l. females were analysed using IMPs-SNP PCR for species identification and detection of *kdr*-*e* and *kdr*-*w* alleles (Table 1). Results showed that 91.43% (IC 95 87.51–94.43%) of specimens were *Anopheles arabiensis*; 5.36% (IC 95 3.03–8.68%) were *Anopheles coluzzii* and 3.21% (IC 95 1.48–6.01%) were *Anopheles gambiae* sensu stricto (s.s.). No hybrid between *An. gambiae* s.s. and *An. coluzzii* was found.

**Table 1:** Allelic frequencies of 1014L, 1014F and 1014S in three sibling species of *Anopheles gambiae* complex in Dielmo

Species	Genotypes						Total (N)	Alleles			2N
	LL	LF	LS	FS	FF	SS		1014L	1014F	1014S	
*An. arabiensis*	128 (0.5000)	2 (0.0077)	28 (0.1081)	19 (0.0742)	10 (0.0391)	69 (0.2664)	256 (1.0000)	286 (0.5586)	41 (0.0801)	185 (0.3613)	512 (1.0000)
*An. coluzzii*	9 (0.6000)	1 (0.0667)	0 (0.0000)	2 (0.1333)	2 (0.1333)	1 (0.0667)	15 (1.0000)	19 (0.6333)	7 (0.2333)	4 (0.1333)	30 (1.0000)
*An. gambiae* s.s.	7 (0.7778)	1 (0.1111)	0 (0.0000)	0 (0.0000)	1 (0.1111)	0 (0.0000)	9 (1.0000)	15 (0.8333)	3 (0.1667)	0 (0.0000)	18 (1.0000)

Figures in parenthesis indicate genotype or allelic frequencies

*N* number of samples characterized, *L* leucine, *F* phenylalanine, *S* serine

The results of genotyping and *kdr* alleles are shown in Table 1. In *An. gambiae* s.s. population, only L1014F mutation was found, at a frequency of 0.1667. Both L1014F and L1014S alleles were found in *An. arabiensis* and *An. coluzzii* populations. The frequency of L1014F allele was 0.233 and 0.080 in *An. coluzzii* and *An. arabiensis*, respectively. However, the frequency of L1014S allele was higher in *An. arabiensis* (0.361) compared to *An. coluzzii* (0.133). Furthermore, individual mosquitoes were observed carrying both *kdr*-*w* and *kdr*-*e* mutations (F/S), with 7.42% (IC 95 4.47–11.22%) and 13.33% (IC 95 1.66–4.05%) in *An. arabiensis* and *An. coluzzii* populations, respectively (Table 1).

**Fig. 5:**
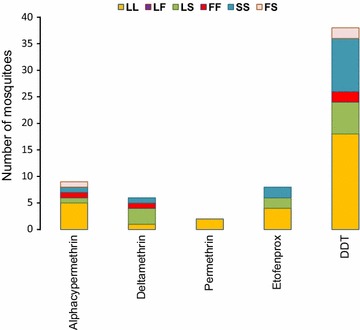
Distribution of susceptible and resistant alleles in *Anopheles arabiensis* individuals alive after exposure to pyrethroids and DDT. *LL* homozygous susceptible, *LF* heterozygous resistant for *kdr*-*w*, *LS* heterozygous resistant for *kdr*-*e*, *FF* homozygous resistant for *kdr*-*w*, *SS* homozygous resistant for *kdr*-*e*, *FS* heterozygous resistant for both *kdr*-*w* and *kdr*-*e*

Among the 280 individuals genotyped, 70 were alive after exposure to insecticides. Of these 70 individuals, 5 were exposed to pirimiphos-methyl and the 65 to pyrethroids and DDT. The genotyping result of 65 individuals exposed was as follows: 95.4% (IC 95 87.10–99.04%) were *An. arabiensis* and 4.6% (IC 95 0.96–12.90%) were *An. coluzzii*. Of the three *An. coluzzii* alive only one was homozygous for L1014F-*kdr* while the two remaining individuals were L1014F and L1014S free. Out of 62 *An. arabiensis* individuals alive, 6.4% (IC 95 1.78–15.70%) were homozygous for L1014F-*kdr* (FF), but there were 22.5% (IC 95 12.03–34.98%) homozygous (SS) and 19.3% (IC 95 10.42–31.37%) heterozygous (LS) individuals for L1014S-*kdr*. Finally, 4.8% (IC 95 1.01–13.50%) of *An. arabiensis* individuals alive were homozygous for both 1014F and 1014S alleles (FS), and 46.8% (IC 95 34.00–59.88%) were fully homozygous susceptible, e.g. L1014F and L1014S free (LL) (Fig. 5). Among *An. arabiensis* individuals alive after exposure to pyrethroids and DDT, some mosquitoes carried both L1014F and L1014S alleles in homozygous (FF, SS) and/or heterozygous (FS, LS) conditions, while others were *kdr*-free individuals. For permethrin, all surviving *An. arabiensis* individuals were homozygous susceptible (LL) (Fig. 5). To get a clear idea of the involvement of *kdr* mutation in *An. arabiensis* susceptibility, an allelic association analysis was performed on 201 individuals, including alive and dead mosquitoes exposed to pyrethroids and DDT (Table 2). No significant correlation was observed, neither between 1014F allele and the resistance to pyrethroids [(OR = 1.17 (0.36–3.88); *p *= 0.78] and DDT [(OR = 1.09 (0.38-3.17); *p *= 0.95], nor between 1014S allele and the resistance to pyrethroids [(OR = 1.38 (0.58–3.30); *p *= 0.46] and DDT [(OR = 1.09 (0.38–3.17); *p *= 0.87] (Table 2).

**Table 2:** Association of *kdr* alleles with pyrethroid and DDT resistance phenotype in *Anopheles arabiensis* in Dielmo

Insecticides (N)	State	Genotype counts						Allelic association (additive model)			
		LL	LF	FF	LS	SS	FS	Fisher exact test (p)		Odds ratio (95% CI)	
								L vs F	L vs S	L vs F	L vs S
Pyrethroids (140)	Alive	11	0	2	6	4	2	(0.787)	(0.463)	1.17 (0.36–3.88)	1.38 (0.58–3.30)
	Dead	64	2	3	8	27	11				
DDT (61)	Alive	20	0	2	6	10	2	(0.953)	(0.873)	1.06 (0.18–6.30)	1.09 (0.38–3.17)
	Dead	10	1	1	3	6	0				

*LL* homozygous susceptible, *LF* heterozygous resistant for *kdr*-*w*, *LS* heterozygous resistant for *kdr*-*e*, *FF* homozygous resistant for *kdr*-*w*, *SS* homozygous resistant for *kdr*-*e*, *FS* heterozygous resistant for both *kdr*-*w* and *kdr*-*e*, *L* leucine, *F* phenylalanine, S serine

## Discussion

In this study, the main issue was to determine insecticide resistance profile, as a backdrop to assessing LLIN vulnerability after a decade of use in Dielmo. In this study, the CDC bottle bioassays have been opted to establish a surveillance tool for detecting resistance to insecticides, but also for providing basic data for vector control routines in Dielmo. The choice for this tool was justified by its adaptability in the field [32], the good correlation of results with those collected from WHO standard assays [37] and its acknowledgement by WHO [35].

From the bioassays, it was observed that *An. gambiae* s.l. was susceptible to lambdacyhalothrin whereas suspected resistance to other pyrethroids including permethrin, deltamethrin, etofenprox, and alphacypermethrin was observed. In Dielmo, LLIN universal coverage started in 2008 and was maintained with three general renewals, in 2011, 2014 and 2016. This long pyrethroid-based vector control could explain the reduced susceptibility to permethrin, deltamethrin, etofenprox, and alphacypermethrin. However, this situation does not appear to affect the downward trend in malaria incidence in Dielmo [29, 38]. In many sites of high or moderate pyrethroid resistance, the effectiveness of LLINs in malaria control has been maintained [29, 38–43] even if this pattern differs from other studies that have shown the failure of LLINs to control pyrethroid-resistant mosquitoes [4, 6, 44]. Also, in Dielmo, *An. gambiae* s.l. was resistant to DDT. *Anopheles* resistance to DDT has often been linked to its historical use for vector-borne diseases and crop pest control. Despite having been abandoned, DDT could persist in the environment due to its decades of widespread use in public health and agriculture. Indeed, DDT was found in high concentrations in many western African plant species, such *Mangifera indica* [45–47], which is also abundant in Dielmo. On the other hand, the Dielmo villagers are engaged in traditional farming and market gardening which could involve use of commercial pesticides comprising organophosphates, organochlorine and pyrethroids. Therefore, selection pressure from agricultural activities, even with low amounts of insecticides, could trigger the development of mosquito resistance to DDT, pirimiphos-methyl and some pyrethroids in Dielmo, as it was observed in many African countries [8, 48–50]. The susceptibility of *An. gambiae* s.l. to bendiocarb and fenitrothion is probably linked to the fact that these molecules are not used for vector control in Dielmo. In this area, the use of LLINs has been the main strategy for controlling malaria vectors for several years, and no IRS programme has been set up for public health purposes. Therefore, the low or almost non-existent selection pressure could explain the full susceptibility of mosquito populations to carbamates, some organophosphates and some pyrethroids.

PCR-based identification of species revealed that *An. arabiensis* was the predominant species within *An. gambiae* complex. The other sibling species, *An. coluzzii* and *An. gambiae* s.s. were found at low levels. This finding is in line with the Dielmo species composition described nearly 20 years ago [51] and recently confirmed despite the implementation of LLINs in this study area [52]. In this study, both 1014F and 1014S alleles were found in *An. arabiensis* and *An. coluzzii* populations while in *An. gambiae* s.s. only1014F was found. This confirms the recent findings on the co-occurrence of these alleles in *An. arabiensis* in Senegal [14] and reinforces the perception that the distribution of *kdr* alleles should no longer be considered as being confined to some specific geographical areas [13, 53]. This is the first report of 1014S (*kdr*-*e*) allele in *An. coluzzii* from Senegal. However, 1014F and 1014S alleles are carried essentially by *An. arabiensis*, which is the principal vector in the study area. In *An. arabiensis*, the frequency of 1014S allele (36%) is in same range as those described in this species from Dakar urban area [14]. The high level of 1014S allele frequency being recorded in Dielmo could mean that the circulation of this allele has been overlooked or underestimated as it was considered until recently that this mutation is absent in western African region. The lack of historical data does not allow estimating when this allele has emerged in Dielmo. However, one cannot rule out that the widespread implementation of LLINs since 2008 has fostered the emergence of *kdr*-*e* mutation. Indeed, it is known that *Anopheles* insecticide resistance can increase considerably over a relatively short period [54].

Furthermore, the frequency of L1014F (*kdr*-*w*) allele in *An. arabiensis* (8%) was relatively low compared to many West African countries [55–59]. All the same, a similar trend was found in *An. arabiensis* population from an area close to Dielmo [60]. Particular attention should be paid to the dynamics of these two alleles as they have not yet approached fixation in the *Anopheles* population and some individuals are carrying both alleles. However, although present in *Anopheles* populations, *kdr* mutation was not strongly associated with phenotype resistance, neither to pyrethroids nor to DDT. Indeed, half of the dead individuals exposed to pyrethroids and DDT were positively genotyped for *kdr* mutation. This indicates that the occurrence of *kdr* mutation is not fully predictive of the resistance to pyrethroids and DDT in *An. gambiae* s.l. from Dielmo. It is thus likely that other mechanisms, in addition to *kdr* mutation, are involved in mosquito resistance to pyrethroids and DDT. This hypothesis was confirmed by use of the synergist EA, implicating GSTs in DDT resistance. Indeed, GST-based resistance is considered the major mechanism of DDT resistance in anopheline species [9, 15, 61, 62] and has been reported in African *An. arabiensis* populations [15, 62]. However, the EA did not fully restore the susceptibility of mosquitoes to DDT; it is therefore likely that additional mechanisms, other than target site (*kdr*) and GSTs, are involved in DDT resistance. It would be relevant to measure esterases and P450s activities, as well as cuticular resistance, to clarify this point.

## Conclusion

In Dielmo, despite a long period of LLIN coverage, the level of pyrethroid resistance in *An. gambiae* s.l. population was still low compared to some malaria-endemic regions in West Africa. Furthermore, to manage insecticide resistance in Dielmo, it is recommended to implement a second line of vector control based on non-pyrethroid/DDT insecticides. However, molecular and biochemical monitoring is needed to better elucidate the different mechanisms involved in insecticide resistance in *Anopheles* populations in Dielmo.

## Abbreviations

ACT: artemisinin-based combination therapy
CDC: Centers for Disease Control and Prevention
CTAB: cetyltrimethyl ammonium bromide
P450s: cytochrome P450 monooxygenases
DDT: dichlorodiphenyltrichloroethane
DEF: *S*-*S*-*S*-tributylphosphorotrithioate
DNA: deoxyribonucleic acid
DT: diagnostic time
EA: etacrynic acid
GSTs: glutathione-*S*-transferases
IMPs: intentional mismatch primers
IRS: indoor residual spraying
*kdr*: knockdown resistance
LLINs: long-lasting insecticide-treated nets
NMCP: National Malaria Control Programme
PBO: piperonyl butoxide
PCR: polymerase chain reaction
SNP: single nucleotide polymorphism
VGSC: voltage-gated sodium channel
WHO: World Health Organization
